# A Cross-Sectional Study Examining the Association between Physical Activity and Perinatal Depression

**DOI:** 10.3390/medicina58091174

**Published:** 2022-08-29

**Authors:** Irene Soto-Fernández, Sagrario Gómez-Cantarino, Benito Yáñez-Araque, Jorge Sánchez-Infante, Alejandra Zapata-Ossa, Mercedes Dios-Aguado

**Affiliations:** 1 Faculty of Physiotherapy and Nursing, University of Castilla-La Mancha, 45004 Toledo, Spain; 2 Department of Nursing, Physiotherapy and Occupational Therapy, Faculty of Physiotherapy and Nursing, University of Castilla-La Mancha, 45004 Toledo, Spain; 3 Department of Business Administration, Faculty of Law and Social Science, University of Castilla-La Mancha, 45071 Toledo, Spain; 4 Performance and Sport Rehabilitation Laboratory, Faculty of Sport Sciences, University of Castilla-La Mancha, 45071 Toledo, Spain; 5 Department of Physical Activity and Sports Sciences, Faculty of Sports Sciences, University of Castilla-La Mancha, Av. Carlos III, s/n, 45071 Toledo, Spain; 6 Yepes Health Center, Health Service of Castilla-La Mancha, Av. Santa Reliquia 26, 45313 Toledo, Spain

**Keywords:** gestational depression, maternal and child health, physical activity, pregnancy

## Abstract

*Background and Objectives:* International organisations recommend that women without illness should have regular moderate-intensity physical exercise throughout their pregnancy and postpartum period as a measure to prevent possible pathologies in both the mother and the newborn. Physical activity during pregnancy reduces the likelihood of depression during pregnancy and after childbirth, benefiting both the pregnant woman and the foetus. However, most pregnant women are known to be inactive. The Pregnancy Physical Activity Questionnaire (PPAQ) analyses the level of physical activity of pregnant women. These data are correlated with the variable depression, for which the Edinburgh Postnatal Depression Scale (EPDS) during pregnancy was used. *Materials and Methods:* The research employed a cross sectional study design on ninety-nine pregnant women. *Results:* The data on physical activity in relation to depression in those pregnant women who had not previously suffered from depression were 719.29 METS min/wk compared with 624.62 METS min/wk in those who had. And for pregnant women who suffered from depression at the time of the study, their physical activity was 698.25 METS min/wk, while those who did not suffer from depression reached 826.57 METS. *Conclusions:* Pregnant women without depression are much more active. A favourable employment situation or a high level of education is directly related to higher physical activity. Physical activity and higher energy expenditure occur at home, as opposed to activity carried out as transport, exercise or at work.

## 1. Introduction

International and national organisations recommend that pregnant women without illness should have regular moderate-intensity physical exercise throughout pregnancy and during the puerperium [1,2] as around 60% of pregnant women are inactive [3]. Hence, the need to prescribe the duration and intensity of physical exercise that a pregnant woman should do [4], since physical exercise is known to prevent diabetes and hypertension [5]. Not forgetting that the practice of physical activity in turn has an impact on child health [6].

The World Health Organisation (WHO) warns that one in four people in their lifetime may suffer from a mental illness, with depression being a very common disorder that can cause sadness, feelings of guilt or shame, loss or interruption of appetite, fatigue and/or lack of concentration [7]. On the one hand, there are studies that support that pregnancy and the postpartum period are protective periods for women against depression due to the lower suicide rate [8], and a recent study conducted during the pandemic underlines the idea that pregnancy has decreased depressive symptoms in this period compared to nonpregnant women [9]. Research carried out in the United States warns that between 8% and 16% of women of reproductive age are affected by this mental pathology [10]. On the other hand, other investigations affirm that the postpartum period increases the risk of MDD (Major Depressive Disorder) [11]. Other studies indicate that women are 70% more likely to suffer from this pathology than men, due to hormonal and/or psychosocial changes experienced by women throughout their lives [12]. However, knowledge is scarce regarding the mechanisms that give rise to mood disorders. Furthermore, studies have not concluded whether increased oestrogen and progesterone affect mood [13,14], or whether it is oestradiol and progesterone that influence levels of the neurotransmitters serotonin, noradrenaline and dopamine that may influence depression [15,16]. In contrast, perinatal mood alterations and psychological factors thought to influence fatigue during gestation have been recognised for more than a century [17,18].

However, some studies show that physical activity during pregnancy reduces the likelihood of depression during pregnancy, and regular physical activity during pregnancy improves the mother’s mood, reduces maternal fatigue and reduces uncertainty about her baby’s health [19,20,21,22]. In addition, yoga-based exercises have been found to have beneficial effects on maternal mood and depression [23]. 

The aim of the present study was to analyse the level of physical activity performed by pregnant women not only as physical exercise, but also when travelling, at home and at work, using the Pregnancy Physical Activity Questionnaire (PPAQ). In addition, the data correlate with the variable depression, so, it was interesting to know whether the depression was pregestational or emerged during pregnancy. The analysis of the variable will be carried out by means of the Edinburgh Postnatal Depression Scale (EPDS) during pregnancy. 

## 2. Materials and Methods

### 2.1. Study Population and Design

The research employed a descriptive, correlational and cross-sectional method [24]. The overall research sample consisted of a total of 99 pregnant women, with a mean age of 32.9 years and a standard deviation of 4.38, who received care throughout their pregnancy at the Health Centre, in a health area of the province of Toledo (Castilla-La Mancha, Spain). In this health area in 2020, according to the municipal census, there were 2570 women, of whom 1040 were of childbearing age (15–49 years). Taking as a reference the 341,315 births in Spain in 2020, the sampling error was 9.85% (confidence level = 95%; z = 1.96; *p* = q = 0.50; α = 0.05). The research was carried out between January 2019 and March 2020. The inclusion criteria were women over 18 years of age, with a singleton pregnancy and who voluntarily agreed to participate in the study. Exclusion criteria were women with pregestational pathologies, such as hypertension, preeclampsia in previous pregnancies or gestational diabetes. 

In addition, the following dependent variables were used for the study: depression before and during pregnancy, age, trimester of pregnancy, level of education, marital status, not having offspring, economic resources and employment status. The independent variables were physical activity at home, physical activity in transport, physical activity at work and total physical activity.

### 2.2. Measuring Instruments 

Two scales were used throughout the research, the first being the semiquantitative questionnaire, Pregnancy Physical Activity Questionnaire (PPAQ) [25]. It is a specific questionnaire of physical activity during pregnancy in which the amount of time the woman is active is recorded. The measurement can be divided into days and reflects the gestational trimester.

The PPAQ is made up of 36 questions, 32 of which refer to the activities carried out by the pregnant woman, including home and/or care of others (13 activities), occupational (5 activities), sports and/or exercise (8 activities), transport (3 activities) and inactivity (3 activities). The Likert scale is used as a response tool, where the response options range from 0 to 5, where 0 means no physical activity at all, 1 less than half an hour a day, 2 from half an hour to almost an hour, 3 from one hour to almost two hours, 4 from two to almost three hours a day and 5 three or more hours a day. 

The PPAQ questionnaire is an easy-to-use measurement tool, as it assigns a value to each activity, expressed in METS, per hour during the week. Thus, according to the intensity of each activity, it is classified as follows: Sedentary (1.5 METs), Light (1.5–3.0 METs), Moderate (3.0–6.0 METs) and High (>6 METS) [26]. The results were analysed following the Guidelines for data processing and analysis of international Physical Activity Questionnaire (IPAQ) [27].

The second scale used was the Edinburgh Postnatal Depression Scale (EPDS) during pregnancy [28,29]. The questionnaire is a validated screening instrument for the detection of depressive symptoms during pregnancy, which consists of 10 items measuring depressive symptoms on a Likert scale, where the scoring options for each item range from 0 to 4. Thus, 0 means not at all, 1 infrequent, 2 sometimes and 3 frequently. So, at the end of the questionnaire, the total response score can range from 0 to 30 points. This means that higher scores indicate a higher probability of suffering from depression. Therefore, this scale is a valid and sensitive instrument for predicting depressive disorders not only in the postnatal but also in the prenatal period.

### 2.3. Statistical Analysis

The data collected were all quantitative, and the statistical package SPSS/PC ver. 25.0 (IBM, Chicago, IL, USA) was used for the analysis. After data collection, the data were transferred to SPSS to create the database needed to study the information collected. Once the database was created, women who did not meet the previously defined inclusion criteria and requirements were removed from the database. Therefore, in turn, a nonparametric correlational study with crosstabulations was carried out. 

Median, mean, standard deviation (SD) or interquartile range (IR) were used to describe the parameters used. Differences in parameter distributions were analysed using Student’s *t*-test, ANOVA or Mann–Whitney U test if the distribution deviated from normality. For categorical data, the chi-square test was used, and relationships between variables were established with Pearson’s or Spearman’s correlation coefficients.

### 2.4. Ethical Considerations

The research was conducted according to the highest ethical standards in accordance with the Declaration of Helsinki, the Guidelines of the International Conference on Harmonisation of Good Clinical Practice and national ethical and legal requirements. All participants signed an informed consent form prior to inclusion in the study, which could be revoked at any time. The Clinical Research Ethics Committee of the Toledo Hospital, Castilla-La Mancha Public Health Service (SESCAM), REC no. 125.

## 3. Results

### 3.1. Characteristics of the Sample

The descriptive analysis of the data reveals the following percentages of age of the pregnant women: 5 (5.1%) of them were between 20–25 years old; 60 (60.6%) were between 26 and 35 years old, and 34 (34.3%) were between 36 and 40 years old. In relation to their gestational trimester, 85 (85.9%) were in the first trimester, and 14 (14.1%) were in the second trimester. Regarding marital status, 80 (80.8%) of the pregnant women reported being married, and 19 (19.2%) reported being single. In terms of offspring, 50 (50.5%) of the participating women have offspring compared to 49 (49.5%) who do not have any. According to the level of studies, 44 (44.4%) of the women in the sample have university studies; 38 (38.4%) have secondary studies, such as baccalaureate or vocational training, and 17 (17.2%) have only primary studies. As far as economic resources are concerned, 74 (74.7%) of the pregnant women said they were unemployed; 15 (15.2%) said they were on sick leave, and 10 (10.1%) were working. 

### 3.2. Physical Activity Carried out by the Pregnant Women in the Study

In order to analyse the physical activity of the women in the sample, the pregnant women were divided into three age groups: (a) 20 to 25, (b) 26 to 35 and (c) 36 to 40 (Table 1).

Once the groups were selected, women in the 20–25 age group had the highest MET score. However, when correlating physical activity and level of education, in all the crosstabulations, it was women with a university education who were more physically active, irrespective of their age group.

However, when crossing the data with the variable employment status, in the first two age groups, women who were working scored higher in METS, while women in the third group scored higher for those who were on sick leave. According to the maximum and minimum scores recorded by the women in the sample in relation to their employment status, the highest scores in all blocks were obtained by working pregnant women, as shown in Table 1.

However, when relating the results of physical activity with age and gestational trimester, in all subgroups, the pregnant women in the second gestational trimester obtained the highest scores: (a) 489.90 METS, (b) 38.50 METS and (c) 161.60.

However, when crossing the data with the variable of having offspring, it was in the 20 to 25 and 26 to 35 age groups that the pregnant women were most active, with the women without offspring achieving the highest figures: group (a) 455.63 METS and group (b) 165.42 METS.

### 3.3. Depression and Physical Activity 

Of the 99 women in the sample, 85.13% reported not having suffered from depression before becoming pregnant, and 14.87% reported having suffered from depression before their pregnancy. 

The results of the PPAQ reveal that pregnant women who had not suffered from depression before pregnancy were more physically active (Table 2). Thus, the maximum score obtained by these women in each block was 1043 METS, 105 METS, 739 METS and 287 METS, respectively. 

With regard to depression during pregnancy, 87.9% of the women in the sample did not show depression when analysing their responses to the EPDS questionnaire, while 12.1% of the women in the sample showed depression when analysing their responses to the EPDS questionnaire. When correlated with the results of the PPAQ, it was observed that the pregnant women who suffered from depression were the women who obtained the highest activity scores (Table 3).

When the sample was tested for normality (Kolmogorov–Smirnov test) with the intention of further statistical analysis, it was observed that the distribution of the sample is not normal. Therefore, a nonparametric analysis was used to test two different groups. When looking at the differences between the results of first trimester pregnant women and second trimester pregnant women, no significant differences were found in either their physical activity or mood. Then, when no significant differences were found, the level of physical activity of the whole sample was analysed to see if it was having an influence. It was found that 58.6% of the pregnant women engaged in light-intensity physical activity, and 41.4% of the pregnant women were sedentary. To understand the data, a crosstabulation was carried out to study the level of physical activity in relation to whether or not they were depressed during pregnancy, but no significant differences were found (Pearson’s chi-square 0.985). When the data on physical activity level were crosstabulated with prepregnancy depression, no significant differences were found (Pearson’s chi-square 0.710).

Therefore, there is no correlation between the results of the Edinburgh scale and physical activity that could serve as a diagnostic tool for perinatal depression. However, there is a significant correlation of 0.570** between the score on the Edinburgh scale and having depression during the first and second trimesters of pregnancy in the women in the sample. The correlation between educational level and employment status was 0.321**. In turn, there was a significant negative correlation between employment status and the Edinburgh scale—0.230*, between employment status and educational level—0.232* and age group with the Edinburgh scale—0.346**.

Finally, a chi-square test was performed to analyse whether there were significant differences between pregnant women who suffered from depression during pregnancy, taking into account whether they had previously developed depression, and the data revealed that there were significant differences (Table 4).

## 4. Discussion

The research study attempts to discover the possible relationship between physical activity and predisposition to suffer from depression during pregnancy and/or the onset of depression during the postpartum period. 

It is known that regular physical activity contributes to good health and reduces the risk of certain concomitant pathologies in pregnancy [3]. In addition, the American College of Obstetrics and Gynecology states that physical activity during pregnancy prevents excessive weight gain during pregnancy, reduces the risk of gestational diabetes, preeclampsia and caesarean delivery. In turn, the British College, the Canadian Society of Obstetrics and Gynaecology and the Spanish Society of Obstetrics and Gynaecology recommend 150 min of moderate physical activity per week throughout pregnancy. Synthesising all this information, the research carried out reveals that the women in the sample put all these physical activity recommendations into practice, as the data show that the average total physical activity carried out was 706.86 METS min/week, with 436.86 METS min/week at home and 436.86 METS min/week at home. Being at home accounted for 436.50 METS, in transport 35.21 METS, as exercise 153.84 METS and at work 82.14 METS. They even put into practice the recommendation proposed by the WHO on physical activity for pregnant women [30], as the pregnant women performed physical activity within the range of 600 to 1500 METS, which corresponds to moderate physical activity, with an average of 706.86 METS.

Studies related to activity in pregnancy show that it is at home that pregnant women have the highest energy expenditure [19,31]. This was confirmed by the research, as the pregnant women in the sample obtained a maximum value of 1043 METS of physical activity at home, regardless of any variable. 

Pregnancy is a complex period of time for both the woman and the new life that is being engendered, as the great transformations that occur throughout gestation can lead to the development of fatigue in the pregnant woman, due to the energy requirements demanded by her foetus and the woman’s weight gain [32]. Thus, these coexisting factors together with the hormonal changes that occur can interfere with the level of physical activity performed by the mother throughout pregnancy, increasing her level of fatigue [15]. This in turn may alter her emotional response to the problems of everyday life, leading to a variation in her mood [33], which is likely to induce the development of perinatal depression [34]. However, of the 99 women in the sample, only 13.1% of the EPDS questionnaire revealed that they suffered from depression during pregnancy, so, the data show that physical activity prevented the development of this pathology. Furthermore, following the data, they found that women who had never suffered from depression were more active than those who had suffered from depression.

However, if we compare all the results obtained in the research with existing studies on the subject, we can see that the studies confirm that mothers who are more physically active suffer less depression and anxiety than mothers who are less physically active [35,36]. 

### Limitations and Future Prospects

In our study, we found that women who had not been depressed prior to pregnancy were the most active, but at the same time, we found that during pregnancy, they suffer a longer period of depression than those who do not do physical activity. The study documented a correlation between the level of physical activity among pregnant women and their depression status. The weakness of the study is its narrow focus on too few variables. The relationship could be related to other variables that contribute to the existence of the relationship (e.g., health conditions or leadership style in the workplace that contribute to the existence of depression [37]). In a complementary study, we could analyse the reasons why women who do physical activity suffer more depression than those who do not, as subjectively we could think that this is due to different external factors that have not been analysed on this occasion, such as greater difficulty in doing physical activity, extreme bodily changes, etc.

On the other hand, it would be necessary to make a more specific selection of the sample having participants between the three trimesters of pregnancy as well as taking into account as a dependent variable the situation of pregnancy at risk. In addition, if it was intended to analyse whether depression in pregnant women is palliated by the practice of physical activity, it would have been convenient to do a good screening of the sample and compare homogeneous and consistent groups of pregnant women with depression and without depression to observe if the activity physical fitness improves the status in women with depression.

In the future, a longitudinal study should be carried out to provide precise data on each woman. We recommend extending the study with a larger representative sample to avoid sampling bias, as well as using other advanced data analysis techniques, for example, Structural Equation Modelling (SEM) or fsQCA. Especially fsQCA allows working with small samples and overcomes some limitations of the dominant research logic [38].

Lastly, it is recommended that a specialist in sports science, in collaboration with the gynaecologist, prescribes physical activity for pregnant women, since benefits have been found for the health of both the mother and the foetus.

## 5. Conclusions

In conclusion, the research reveals that regular physical activity is beneficial for the women in the study. In relation to depression, pregnant women without depression are much more active. In addition, having a favourable employment situation or a high level of education is directly related to being more physically active when pregnant. Additionally, the results show that the greatest energy expenditure and the greatest amount of physical activity are carried out by pregnant women at home.

## Figures and Tables

**Table 1:** MET scores by activity block and age group.

		N	Mean	Standard Deviation	Standard Error	95% Confidence Interval for the Mean	
						Lower Limit	Upper Limit
MET Home	20 to 25 years old	5	597.38	276.86	123.82	253.61	941.15
	26 to 35 years old	60	433.16	146.98	19.14	394.86	471.46
	36 to 40 years old	34	417.61	173.99	29.84	356.90	478.32
	Total	99	436.50	166.04	16.69	403.40	469.61
MET Transport	20 to 25 years old	5	49.00	31.30	14.00	10.13	87.87
	26 to 35 years old	60	34.88	18.04	2.35	30.18	39.58
	36 to 40 years old	34	33.97	14.64	2.51	28.86	39.08
	Total	99	35.21	17.76	1.79	31.67	38.75
MET Exercise	20 to 25 years old	5	234.08	160.15	71.62	35.23	432.93
	26 to 35 years old	60	145.74	140.80	18.33	109.05	182.44
	36 to 40 years old	34	158.57	79.69	13.67	130.77	186.38
	Total	99	153.84	123.96	12.46	129.12	178.57
MET Work	20 to 25 years old	5	119.14	102.09	45.66	−7.62	245.90
	26 to 35 years old	60	79.53	76.61	9.97	59.56	99.49
	36 to 40 years old	34	82.09	45.21	7.87	66.06	98.12
	Total	99	82.14	68.54	6.92	68.40	95.88
PPAQ	20 to 25 years old	5	999.60	476.29	213.00	408.21	1590.99
	26 to 35 years old	60	693.31	315.25	41.04	611.15	775.46
	36 to 40 years old	34	689.83	187.55	32.16	624.39	755.27
	Total	99	706.86	290.82	29.23	648.85	764.86

**Table 2:** PPQA results for the variable previous depression.

		N	Mean	Standard Deviation	Standard Error	95% Confidence Interval for the Mean	
						Lower Limit	Upper Limit
MET Home	No	86	445.12	168.53	18.17	408.98	481.25
	Yes	13	379.45	141.09	39.13	294.19	464.71
	Total	99	436.50	166.04	16.69	403.38	469.61
MET Transport	No	86	35.33	18.22	1.97	31.42	39.23
	Yes	13	34.46	14.98	4.15	25.41	43.51
	Total	99	35.21	17.76	1.79	31.67	38.75
MET Exercise	No	86	155.71	126.41	13.63	128.61	182.81
	Yes	13	141.51	110.02	30.52	75.02	207.99
	Total	99	153.84	123.96	12.46	129.12	178.57
MET Work	No	86	84.12	70.81	7.68	68.84	99.39
	Yes	13	69.19	51.58	14.31	38.02	100.36
	Total	99	82.14	68.54	6.92	68.40	95.88
PPAQ	No	86	719.29	299.23	32.27	655.14	783.45
	Yes	13	624.62	219.25	60.81	492.12	757.11
	Total	99	706.86	290.82	29.23	648.85	764.86

**Table 3:** PPQA scores for depression during pregnancy.

		N	Mean	Standard Deviation	Standard Error	95% Confidence Interval for the Mean	
						Lower Limit	Upper Limit
MET Home	No	87	432.79	158.97	17.04	398.91	466.67
	Yes	12	463.34	217.14	62.68	325.38	601.31
	Total	99	436.50	166.04	16.69	403.38	469.61
MET Transport	No	87	34.84	16.88	1.81	31.24	38.44
	Yes	12	37.92	23.96	6.92	22.69	53.14
	Total	99	35.21	17.76	1.79	31.67	38.75
MET Exercise	No	87	147.02	116.52	12.49	122.19	171.86
	Yes	12	203.29	166.45	48.05	97.53	309.05
	Total	99	153.84	123.96	12.46	129.12	178.57
MET Work	No	87	77.27	63.46	6.84	63.66	90.87
	Yes	12	117.02	93.74	27.06	57.45	176.58
	Total	99	82.14	68.54	6.92	68.40	95.88
PPAQ	No	87	691.04	267.82	28.71	633.96	748.12
	Yes	12	821.57	420.02	121.25	554.70	1088.43
	Total	99	706.86	290.82	29.23	648.85	764.86

**Table 4:** Differences between women with depression before and during pregnancy and those who did not.

Chi-square	53.828	56.818
Df ^1^	1	1
Asymptotic sig.	0.000	0.000

^1^ df = Degree of freedom.

## Data Availability

The data that support the findings of this study are available from the corresponding author upon request.
